# Waste Valorization: Effective Removal of Methylene Blue by Magnetic Composite Biochar Prepared from Red Mud and Medulla Tetrapanacis

**DOI:** 10.3390/ma19204292

**Published:** 2026-10-12

**Authors:** Fuming Jia, Hongqiang Song, Muyang Tao, Wei Su, Yunrui Zhao, Zhou Wang, Jie Wu, Qiancheng Ding, Hui Luo, Baojie He

**Affiliations:** 1 School of Civil and Ocean Engineering, Jiangsu Ocean University, Lianyungang 222005, China; j2669016820@163.com (F.J.); shq@jou.edu.cn (H.S.); m591652061@163.com (M.T.); 19502520235@163.com (W.S.); zhaoyr997997@163.com (Y.Z.); 18952043929@163.com (J.W.);; 2 Centre for Climate-Resilient and Low-Carbon Cities, School of Architecture and Urban Planning, Chongqing University, Ministry of Education, Chongqing 400045, China

**Keywords:** red mud, waste valorization, magnetic biochar, pyrolysis, methylene blue, adsorption

## Abstract

Removing hazardous dyes from wastewater is essential for protecting the environment and reducing health risks. In this study, a magnetic composite biochar was successfully prepared via a pyrolysis method using industrial solid waste red mud and agricultural by-product Medulla tetrapanacis as raw materials, and its adsorption performance toward methylene blue (MB) was systematically evaluated. Among the prepared composites, MRMT exhibited superior removal capacity, with a Langmuir maximum adsorption capacity of 285.2 mg/g. The adsorption process followed the pseudo-second-order kinetic model and the Langmuir isotherm, indicating monolayer adsorption. Thermodynamic analysis gave ΔH^0^ = 19.691 kJ/mol and ΔS^0^ = 214.335 J/(mol·K), suggesting that the adsorption process was spontaneous and endothermic. Comprehensive characterization by XRD, FTIR, and XPS revealed that MB removal involved electrostatic attraction, π–π stacking, n–π interactions, and hydrogen bonding. MRMT exhibited a significant magnetic response (13.66 emu/g) and maintained an adsorption capacity of over 57% after five cycling experiments, demonstrating good recyclability. Even in the presence of coexisting ions (Pb^2+^, Cr^3+^, Ni^2+^, Cu^2+^, Zn^2+^), it still retained a relatively high adsorption efficiency. The research findings indicate that MRMT, prepared from solid waste red mud and the agricultural by-product Tongcao, is an adsorbent that is easily recovered magnetically; both types of raw materials have the potential for low-cost utilization and can provide valuable technical insights for dye wastewater treatment.

## 1. Introduction

Water pollution, especially dye contamination in industrial wastewater, has become an increasingly severe environmental issue worldwide, posing a serious threat to aquatic ecosystems and human health [1]. Dye wastewater is characterized by intense color, high toxicity, and resistance to degradation. If discharged directly without effective treatment, it can cause irreversible damage to the environment and may pose potential mutagenic or carcinogenic risks to organisms [2]. Methylene blue (MB), as a cationic dye widely used in the textile, papermaking, plastics, and pharmaceutical industries, has brought convenience to human needs; however, its toxicity and resistance to natural degradation processes pose a serious threat to aquatic ecosystems and human health [3]. Therefore, the development of efficient, economical, and environmentally friendly technologies for dye removal from wastewater has become a research hotspot in the field of environmental science.

At present, advanced oxidation processes (AOPs) [4], membrane separation technologies [5], biological methods [6], and adsorption [7] are the main technical approaches for MB removal from wastewater. Among them, adsorption is considered one of the most commonly used techniques for removing aquatic pollutants due to its advantages of simple operation, cost-effectiveness, high treatment efficiency, and wide applicability. The adsorption process involves an interaction between dye molecules and the adsorbent surface, and its efficiency is significantly influenced by adsorbent properties and environmental conditions such as pH, temperature, initial dye concentration, and adsorbent dosage [8]. Biochar, as a new adsorption material, is characterized by rich surface functional groups, high specific surface area, developed pore structure and environmental friendliness [9]. Biochar is a low-density carbonaceous material produced by pyrolysis of biomass under oxygen-limited conditions and has been proven to be an ideal adsorbent for the removal of various pollutants from aqueous solutions, including heavy metals, organic pollutants, and dyes [10].

However, conventional biochar still faces some challenges in practical applications, such as difficulties in recovery and the need for improved adsorption selectivity. To overcome these limitations, researchers have begun to explore the combination of biochar with magnetic materials to prepare magnetic biochar composites. Magnetic biochar not only inherits the excellent adsorption capacity of biochar but is also endowed with the ability to be rapidly separated and recovered under an external magnetic field [11], thereby greatly simplifying the post-treatment process and reducing operating costs. In addition, the adsorption performance and selectivity toward specific pollutants can be further enhanced by introducing specific components into biochar [12].

Red mud (RM) is a strongly alkaline industrial solid waste generated during the Bayer process for alumina production [13], with a global annual output exceeding 150 million tons, yet its comprehensive utilization rate is less than 10%. Its direct stockpiling occupies a large amount of land and causes environmental pollution [14]. However, red mud is rich in iron oxides (such as hematite and magnetite), making it a potential source for the preparation of magnetic materials [15]. The application of red mud in adsorbent preparation not only enables recovery of solid waste and reduces material costs but also provides the convenience of magnetic separation. Medulla tetrapanacis (MT) is the dried pith of *Tetrapanax papyrifer* (Hook.) K. Koch, which grows in southern and southwestern China. After pyrolysis, it exhibits a porous structure [16], with wide availability and low cost, showing potential for biochar production. Moreover, MT demonstrates good affinity for pollutants, which may be related to its porous structure and abundant surface chemical functional groups [17]. The combination of MT biomass with red mud is expected to develop a novel, efficient, and easily recoverable adsorbent. For example, Li et al. prepared a composite biochar from red mud and food waste compost, which served as an efficient adsorbent for MB, with an adsorption capacity of 124.06 mg/g determined by the Langmuir isotherm model [18]. Qiu et al. prepared a magnetic adsorbent using red mud and peanut shells, achieving a maximum MB adsorption capacity of 123.26 mg/g [19].

This study aims to explore the preparation of a magnetic recyclable composite biochar from Medulla tetrapanacis and red mud via a pyrolysis method [20] and to systematically investigate its adsorption performance toward methylene blue. By means of SEM, BET, XRD and FTIR, the microstructure and physical and chemical properties of the composite biochar were systematically explored, and the adsorption performance and mechanism toward methylene blue were thoroughly discussed in combination with adsorption kinetics, adsorption isotherms, and thermodynamic analysis. In addition, the effects of environmental factors such as pH and coexisting ions on the adsorption process were examined, and the regeneration performance of the adsorbent was evaluated. The sourcing of Tongcao and red clay used in this study offers potential for low-cost production and enables recycling of solid waste, providing a theoretical basis and experimental support for the design and development of novel high-efficiency adsorbent materials.

## 2. Materials and Methods

### 2.1. Materials and Reagents

The hydrochloric acid (HCl), sodium hydroxide (NaOH) standard titration solutions, metal nitrates and methylene blue dye (MB, C_16_H_18_ClN_3_S·3H_2_O, purity ≥ 98.5%) used in this study were purchased from Shanghai Guoyao Chemical Reagents Co., Ltd., Shanghai, China. All MB solutions used in the experiments were prepared by directly weighing the trihydrate form of the reagent and dissolving it in deionized water. The pH of the solutions was adjusted using standard HCl and NaOH titration solutions. Industrial solid waste red mud was obtained from Chalco Shandong Co., Ltd. (Shandong, China).

The collected red mud samples were washed and crushed by a jaw crusher (PEX 150 × 250, Henan Ford Machinery Co., Ltd., Zhengzhou, China) to obtain granular materials with a particle size of 0.5–5 mm. The crushed particles were placed in a ball mill (MQGg1212, Henan Ford Machinery Co., Ltd., Zhengzhou, China) for 2 h. After grinding, the red mud was washed repeatedly with deionized water until the filtrate was neutral, placed in an oven and dried at 80 °C, then manually ground and screened by a 100 μm sieve to prepare red mud powder, denoted as RM. The agricultural by-product Medulla tetrapanacis (MT) was purchased from Guangyuan Lintai Traditional Chinese Medicine Development Co., Ltd. (Guangyuan, China). It was washed with deionized water and dried at 80 °C to remove residual moisture. After complete drying, the MT was ground in a ball mill for 2 h and then passed through standard sieves with different mesh sizes to obtain MT powder with a particle size less than 2.0 mm, denoted as MT.

### 2.2. Preparation of Composite Biochar

**Figure 1:**
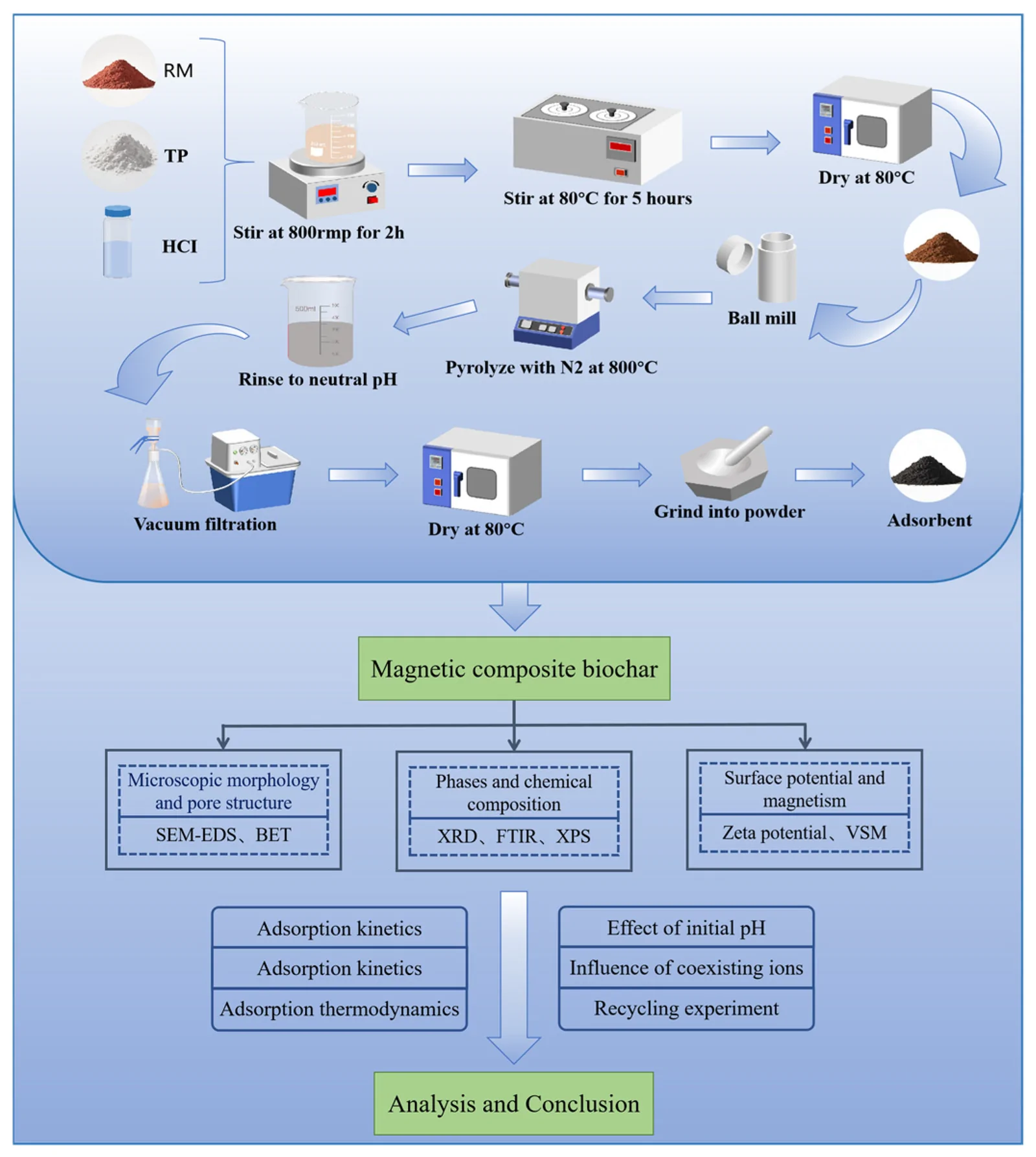
Schematic diagram of the experimental procedure.

We mixed 10 g of RM and MT in mass ratios of 0:10, 3:7, 5:5, 7:3, and 10:0. First, RM was added to a beaker containing 50 mL of HCl (1 mol/L) solution and stirred at 200 rpm on a magnetic stirrer to achieve uniform dispersion, after which MT was slowly added, and the mixture was continuously stirred at 800 rpm for 2 h to ensure thorough mixing. The beaker was then placed on a thermostatic stirrer and stirred at 80 °C and 500 rpm for 5 h, followed by drying in an oven at 80 °C. The mixture was pyrolyzed in a tube furnace (KSL-1100X, Hefei Kejing Materials Technology, Hefei, China) after being purged with high-purity N_2_ at a flow rate of 0.5 L/min to create an inert atmosphere. It was heated at a rate of 10 °C/min to the target temperature of 800 °C, held at that temperature for 2 h, and then allowed to cool naturally to ambient temperature. The biochar samples were washed with deionized water until neutral, filtered, and dried in an oven at 80 °C. After drying, the samples were ground and passed through a 150 μm sieve. Finally, the prepared materials were stored in a vacuum desiccator. The mixed samples were labeled PMT (pyrolyzed Medulla tetrapanacis), LRMT, MRMT, HRMT, and PRM (pyrolyzed red mud) according to increasing RM content. The experimental procedure is shown in Figure 1.

### 2.3. Material Characterization

Using an FEI QUANTA 250G environmental scanning electron microscope (SEM, FEI Company, Hillsboro, OR, USA) and an energy dispersive spectrometer (EDS, EDAX Inc., Pleasanton, CA, USA), the difference in element composition between the samples before and after modification was compared. With the help of a Micromeritics TriStar II Plus automatic specific surface area and pore size analyzer (BET, Micromeritics Instrument Corporation, Norcross, GA, USA), the pore size distribution characteristics of the samples were tested. The zeta potential of the adsorbent in the aqueous phase system was characterized by a zeta potential analyzer (Anton Paar, Graz, Austria). In the crystal phase structure test, an X-ray diffractometer (XRD, JEOL Ltd., Tokyo, Japan) was selected. A Fourier transform infrared spectrometer (FTIR, Thermo Fisher Scientific, Waltham, MA, USA) was used to analyze the functional groups on the surface of the samples. The binding energy of the sample elements was collected by a JEOL JPS-9200 X-ray photoelectron spectrometer (XPS, JEOL Ltd., Tokyo, Japan). A VSM-5000 vibrating sample magnetometer (Nanbei Instruments, Zhengzhou, China) was used to measure the magnetic properties of the adsorbent. The ion concentration of the solution was determined using an iCAP 7000 Inductively Coupled Plasma Optical Emission Spectrometer (ICP-OES, Thermo Fisher Scientific GmbH, Bremen, Germany). The concentration of methylene blue (MB) was determined by a TU-1810 ultraviolet–visible spectrophotometer (UV–Vis, Beijing Purkinje General Instrument Co., Ltd., Beijing, China).

### 2.4. Adsorption Tests

A series of adsorption experiments was carried out to investigate the influence of different conditions and parameters on adsorption behavior. Adsorption time (5–480 min), initial MB dye concentration (60–300 mg/L), adsorption temperature (288–308 K), and pH (2–10) were investigated through adsorption experiments. Prior to the addition of the adsorbent, 0.1 mol/L HCl and 0.1 mol/L NaOH solutions were used to fine-tune the pH of the methylene blue (MB) solution to ensure uniform pH conditions in the system. In each experiment, 50 mg adsorbent was added to 100 mL MB solution.

After the adsorption experiments were completed, solid–liquid separation was carried out using a 0.45 μm filter membrane in order to remove any suspended particles that had not settled in the solution. Ultraviolet–visible spectrophotometry was employed with a characteristic absorption wavelength of 664 nm to quantitatively analyze the remaining amount of MB in the filtrate. For all adsorption experiments in this study, three parallel samples were used to reduce experimental errors; the final data are presented as averages ± standard deviations. Using MB solutions at concentrations of 10 mg/L, 50 mg/L, and 100 mg/L (each solution paired with a specific cationic salt solution at concentrations of 10 mg/L, 50 mg/L, and 100 mg/L, respectively, in a 1:1 ratio), the effects of coexisting ions (including Pb^2+^, Cr^3+^, Ni^2+^, Cu^2+^, and Zn^2+^) were evaluated in both single-ion and mixed-ion systems. In all coexisting ion experiments, stock solutions were prepared using metal nitrates (with NO^3−^ as the counterion).

The equilibrium adsorption capacity (Q_e_, mg/g) and the MB removal efficiency (R_t_, %) were obtained from Equations (1) and (2), respectively.

Q_e_ = [(C_0_ − C_e_)/m] × V (1)

R_t_ = [(C_0_ − C_e_)/C_0_] × 100% (2)

The adsorption capacity at any given time (Q_t_) was obtained from Equation (3):

Q_t_ = [(C_0_ − C_e_)/m] × V (3)

where C_0_ (mg/L) and C_e_ (mg/L) are the initial and equilibrium concentrations of MB, respectively, and V (L) and m (g) are the volume of the MB solution and the mass of the adsorbent, respectively.

During the kinetic adsorption experiments, 50 mg of the adsorbent was transferred into an Erlenmeyer flask containing the MB solution. This was performed under neutral conditions; no additional pH adjustment was required. The flask was then placed in a thermostatic shaker and agitated at 150 rpm at 25 °C. Each test used 100 mL of MB solution with an initial concentration of 100 mg/L, obtained by diluting the stock solution at a ratio of 1:10, and a fixed dose of 50 mg of adsorbent was added to each sample. After predetermined time intervals (5–480 min), the MB concentration in the solution was determined. The pseudo-first-order (PFO) and pseudo-second-order (PSO) kinetic models and the intraparticle diffusion model were applied to effectively elucidate the adsorption mechanism. Their linearized equations are as follows:

log(Q_e_ − Q_t_) = log Q_e_ − k_1_t/2.303 (4)

(5) $$t/Q_{t}=1/k_{2}Q_{e}^{2}+t/Q_{e}$$

Q_t_ = K_id_t^1/2^ + C (6)

where Q_t_ (mg/g) is the adsorption capacity at time t (min) and Q_e_ is the adsorption capacity at equilibrium. The pseudo-first-order and pseudo-second-order rate constants are denoted by K_1_ (1/min) and K_2_ (g/(mg·min)), respectively. K_id_ is the intraparticle diffusion rate constant (mg/(g·min^0.5^)), and C is directly proportional to the boundary layer thickness (mg/g).

In the adsorption isotherm experiments, 50 mg of the adsorbent was added to MB solutions with different initial concentrations (60–220 mg/L). As this was performed under neutral conditions, no additional pH adjustment was required, and the other conditions were kept the same as described above. The equilibrium adsorption capacity (Q_e_) and equilibrium concentration (C_e_) were calculated. The corresponding model equations were used to fit and analyze the experimental data. Equations (7) and (8) are nonlinear expressions of the Langmuir and Freundlich models, respectively.

Q_e_ = Q_max_K_L_C_e_/(1 + K_L_C_e_) (7)

(8) $$Q_{e}=K_{F}C_{e}^{1/n}$$

R_L_ = 1/(1 + K_L_C_0_) (9)

where Q_max_ represents the maximum adsorption capacity of the adsorbent. K_L_ (L/mg) is the Langmuir constant, which reflects the affinity between the adsorbent and adsorbate. K_F_ is the Freundlich constant, which indicates the adsorption intensity and the adsorption capacity of the adsorbent. n is the Freundlich heterogeneity index, which is used to describe the unevenness of the adsorbent surface. When n > 1, it means that the adsorption process is easy to carry out. The equilibrium parameter R_L_ can be used to characterize the adsorption characteristics of the Langmuir model.

In order to explore the influence of temperature on the adsorption behavior of materials, three temperature gradients of 288 K, 298 K and 308 K were selected to systematically analyze the effect of temperature on the adsorption capacity of the adsorbents. By calculating and analyzing thermodynamic parameters, the spontaneity and thermodynamic characteristics of the adsorption process were assessed, such as the Gibbs free energy change (ΔG^0^, kJ/mol) (Equation (10)), enthalpy change (ΔH^0^, kJ/mol), and entropy change (ΔS^0^, J/(mol·K)) (Equation (11)).

ΔG° = −RT ln K_d_ (10)

(11) $$\ln K_{d}=\frac{\Delta S{}^{\circ}}{R}-\frac{\Delta H{}^{\circ}}{\mathrm{RT}}$$

K_d_ = K_L_M_0_C^0^ (12)

where R is the gas constant (8.314 J/(mol·K)), T is the absolute temperature (K), K_d_ is the dimensionless thermodynamic equilibrium constant, M_0_ is the molar mass of MB (373.90 g/mol) and C^0^ is the molar concentration of water (55.5 mol/L) [21].

### 2.5. Recovery Capacity

In this study, the reuse and regeneration performance of the adsorbent were systematically evaluated through five consecutive adsorption–desorption cycle tests. In each cycle experiment, 50 mg of the adsorbent was taken and added to the methylene blue solution with a concentration of 100 mg/L to complete the adsorption reaction. The volume of the solution was 100 mL, and the contact time was 480 min. After adsorption, the recovered adsorbent was desorbed using ethanol, and the sample was fully washed with deionized water to remove residual impurities and eluent on the surface. Then, after drying at 80 degrees Celsius, it was used in the next cycle of testing, so as to complete repeated regeneration experiments. We used “cycle 0” to represent the initial adsorption experiment before regeneration.

## 3. Results

### 3.1. Characterization of Materials

**Figure 2:**
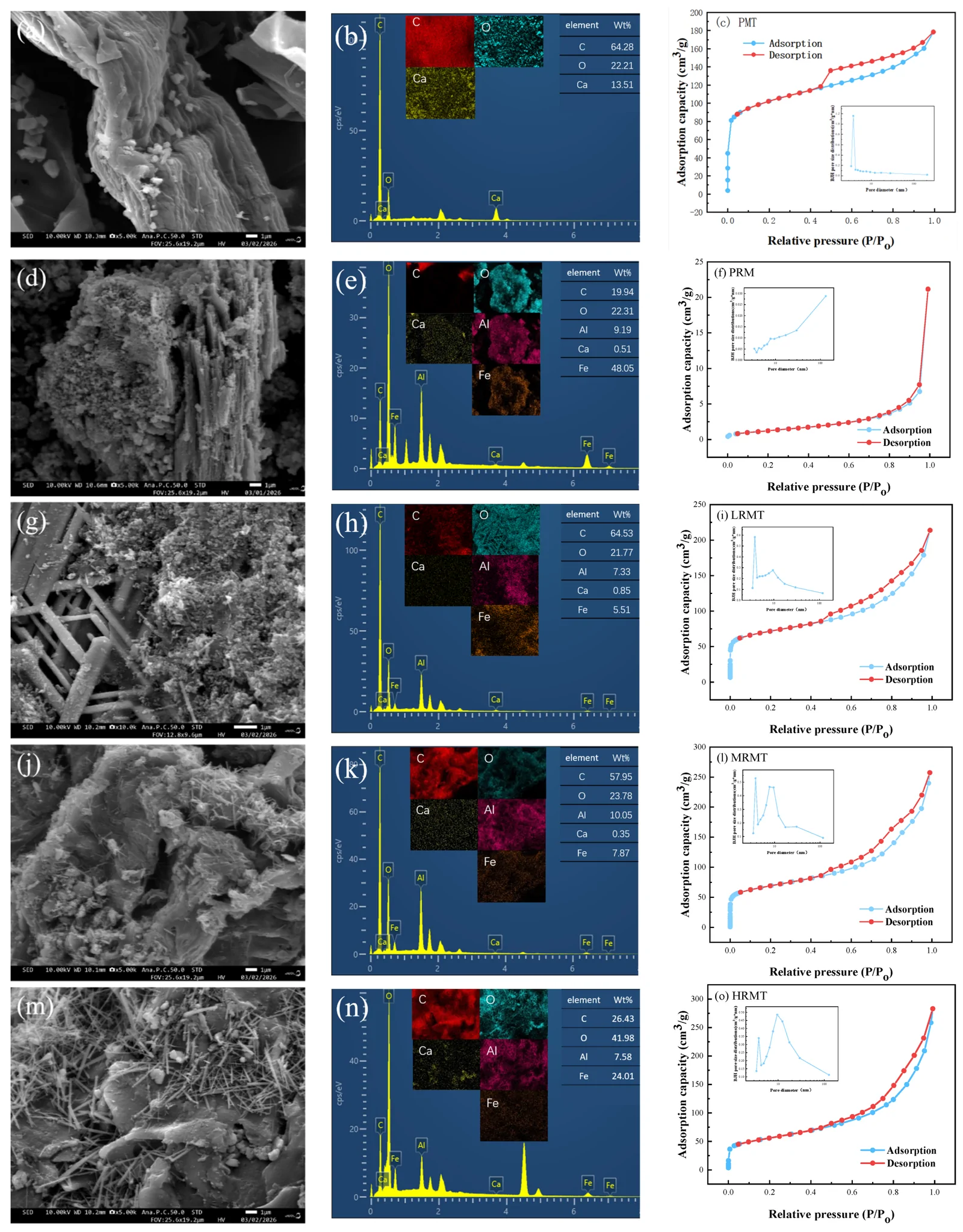
SEM images, EDS elemental data, and N_2_ adsorption–desorption isotherms and pore size distributions of PMT (**a**–**c**), PRM (**d**–**f**), LRMT (**g**–**i**), MRMT (**j**–**l**), and HRMT (**m**–**o**).

Figure 2 systematically presents the SEM micro-morphology, EDS elemental distribution, and N_2_ adsorption-desorption pore structure characteristics of PMT, PRM, and the composite biochars with different mixing ratios. As shown in Figure 2a, PMT exhibits a lamellar structure with distinct wrinkle textures on the surface, and numerous fine particles are uniformly attached, which can provide abundant active sites for adsorption. Within the detection limits of EDS, Figure 2b reveals that PMT contains only three elements, namely, C, O, and Ca, with a carbon mass fraction as high as 64.28%, and no active metals such as Fe or Al are detected. The Ca originates from the natural carbonates within the MT tissue, and its elemental mapping completely overlaps with the carbon matrix, confirming that PMT is a pure carbon-based material. In contrast, PRM (Figure 2d) consists of flocculent aggregates formed by the close agglomeration of iron–aluminum oxide nanoparticles, exhibiting a loose and rough morphology. PRM (Figure 2e) is dominated by O and Fe, with the signals of Fe (48.05%) and Al (9.19%) being highly consistent with the mineral agglomeration regions, confirming that the matrix is composed of iron–aluminum oxides [22], while the trace amount of carbon originates solely from residual organic matter. It should be noted that EDS is a semi-quantitative micro-area characterization technique and does not represent the precise elemental composition of the sample as a whole.

After compounding the two raw materials, the surface of LRMT (Figure 2g) exhibits a uniform dispersion of a small amount of RM particles, with a clear grid-like fibrous structure that provides channels for pollutant transport. For MRMT (Figure 2j), the surface of the lamellar carbon matrix is covered with numerous acicular microcrystals, resulting in a rough and highly porous surface that effectively increases the number of active adsorption sites. In HRMT (Figure 2m), with the increased proportion of RM, abundant needle-like crystals are precipitated and interwoven, which may cause local crystal stacking and consequently lead to pore blockage. All three composite biochars possess rough surfaces, which can provide abundant active sites for subsequent adsorption [23]. By comparing the EDS results in Figure 2h,k,n in sequence, it can be observed that the elemental concentrations in the three samples increased or decreased accordingly, which corresponds to the increase in red mud content during the preparation process.

As shown in Figure 2c,f, the N_2_ adsorption–desorption isotherm of PMT is classified as type IVa with a typical H3 hysteresis loop [24]. The BET specific surface area is 374.8 m^2^/g, and the total pore volume is 0.2760 cm^3^/g, as shown in Table 1, indicating that PMT is predominantly mesoporous [25]. The pore sizes of PMT are mainly concentrated in the range of 2–50 nm, further confirming its mesoporous nature. As shown in Figure 2f, PRM belongs to a type IVa isotherm with an H3-type hysteresis loop, with a BET specific surface area of 4.707 m^2^/g and a total pore volume of 0.0327 cm^3^/g, indicating that the pore structure of PRM is mainly composed of macropores [26], which is consistent with the SEM observations. In addition, the abundant pore structure facilitates better access of MB molecules into the interior of the adsorbent through pore filling [27].

Comparing Figure 2i,l,o, the isotherms of the three composite biochars, LRMT, MRMT, and HRMT, are all classified as type IVa, indicating the formation of a unique mesoporous framework within the composite biochars [28]. As the RM doping ratio increased, the BET specific surface area and total pore volume of the composite biochars continuously decreased, while the average pore size gradually increased. The porous structure provides mass transfer pathways for the diffusion of methylene blue molecules, facilitating the migration of pollutants into the adsorbent; however, in subsequent studies, the adsorption performance did not show a simple positive correlation with BET specific surface area. The BET specific surface area of MRMT (248.5 m^2^/g) was slightly lower than that of LRMT (262.2 m^2^/g), but its maximum Langmuir adsorption capacity was actually higher; after normalizing the adsorption capacity by specific surface area, the adsorption capacity per unit area was HRMT > MRMT > LRMT, which is exactly the opposite of the order based on BET specific surface area. This phenomenon indicates that a material’s adsorption capacity is not determined by surface area alone; rather, surface chemical properties play a dominant role. The active Fe/Al mineral sites, oxygen-containing functional groups, and residual calcium-containing mineral phases introduced by red mud may be key factors enhancing the adsorption capacity of methylene blue. Excessive red mud doping (HRMT) leads to agglomeration of inorganic mineral particles and microporous blockage, which not only reduces the specific surface area but also masks some of the active surface sites, ultimately resulting in a decline in adsorption performance.

### 3.2. Adsorption Kinetics

**Figure 3:**
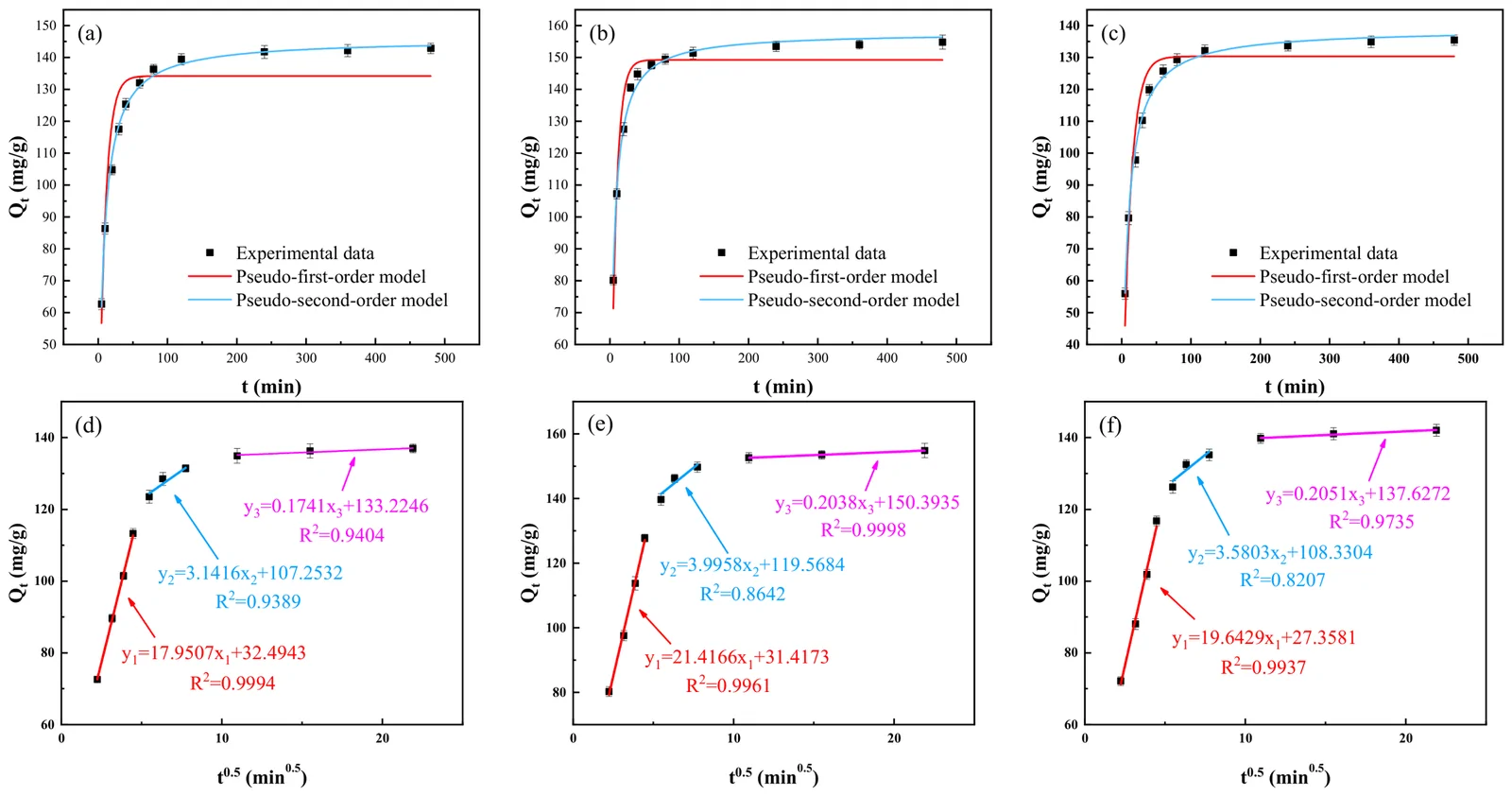
PFO and PSO kinetic models: (**a**) LRMT, (**b**) MRMT, and (**c**) HRMT. Intraparticle diffusion model: (**d**) LRMT, (**e**) MRMT, and (**f**) HRMT.

To further understand the adsorption process, kinetic studies were carried out on the composite biochars. This study aimed to reveal the adsorption rate and mechanism of MB on the adsorbent surfaces under identical experimental conditions. As shown in Figure 3a–c, the kinetic curves indicate that the adsorption of MB onto LRMT, MRMT, and HRMT exhibited similar trends, with a sharp increase in adsorption capacity within the first 100 min, followed by a gradual approach to equilibrium. Notably, MRMT exhibited the highest equilibrium adsorption capacity (157.9 mg/g), markedly higher than those of LRMT (145.7 mg/g) and HRMT (139.0 mg/g). This phenomenon may be due to the fact that at a 1:1 mixing ratio, the density of active sites in the Fe/Al minerals introduced by the red mud reaches an optimal level, thereby enabling MRMT to achieve optimal adsorption performance. The fitting parameters for the adsorption kinetics of the three adsorbents are shown in Table 2. By comparing the correlation coefficients obtained from each kinetic model, it can be seen that the determination coefficient R^2^ for the quasi-second-order kinetic model ranges from 0.992 to 0.997, which is markedly higher than the 0.937 to 0.943 value for the quasi-first-order kinetic model. This indicates that the adsorption process is better described by the quasi-second-order kinetic model [29].

To further elucidate the diffusion-controlled mechanism, the intraparticle diffusion model was applied for piecewise fitting, as shown in Figure 3d–f. The adsorption process of MB onto the three adsorbents can be divided into three typical stages. The first stage is controlled by external diffusion, during which MB molecules rapidly cross the liquid film boundary layer and adsorb onto the external surface of the particles. The second stage is dominated by intraparticle diffusion, where molecules migrate along the pore channels toward the internal micropores; the diffusion rate decreases significantly due to pore tortuosity and size limitations. The third stage approaches equilibrium, where most of the internal and external active sites are occupied, the concentration gradient approaches zero, and the diffusion rate further declines. In addition, the piecewise linear plots for all three samples did not pass through the origin, indicating that intraparticle diffusion is not the sole rate-limiting step throughout the entire adsorption process; rather, both film diffusion and intraparticle diffusion jointly govern the overall adsorption rate [30].

### 3.3. Adsorption Isotherm

**Figure 4:**
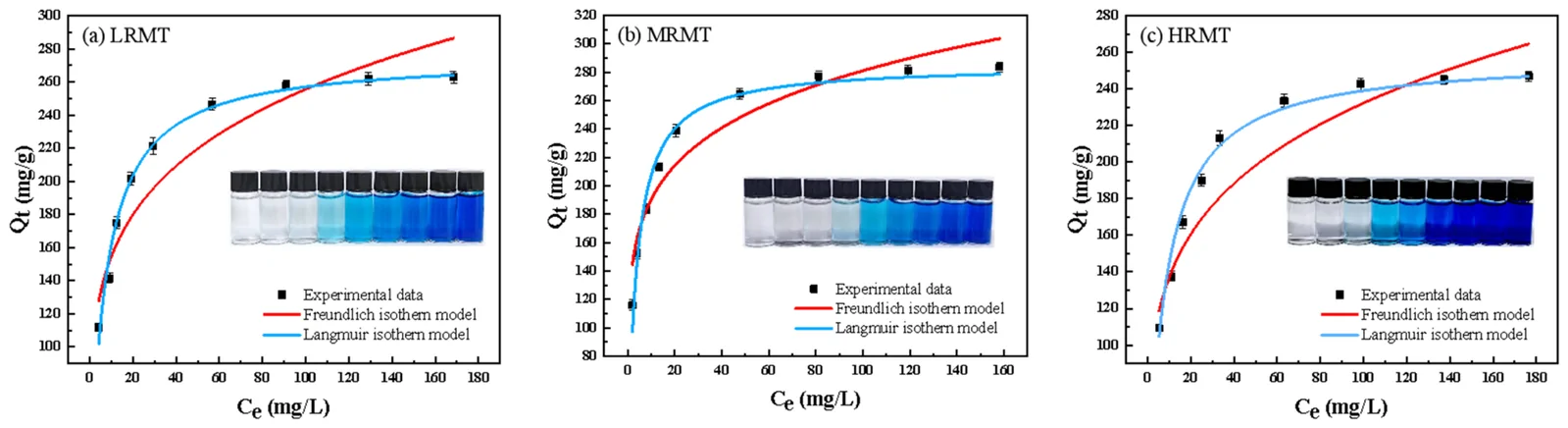
Adsorption isotherms of LRMT, MRMT, and HRMT.

According to the analysis in Figure 4, the initial concentration of methylene blue (MB) has a significant influence on the adsorption performance of composite biochar. Under the condition of low initial concentration, the adsorption capacity of the adsorbent for MB increases rapidly with the increase in solution concentration. When the concentration of MB dye is further increased, the growth rate of the adsorption capacity of the three adsorbents gradually decreases and finally tends to a saturated and stable state, and the corresponding dye removal rate also shows a gradual decline. The mass transfer driving force generated by the concentration gradient is an important factor promoting the adsorption reaction, and a higher initial dye concentration provides sufficient driving force for the adsorption process, which is the main reason for the rapid increase in adsorption capacity at low concentration ranges [31]. In contrast, when the isotherm curves tend to flatten, it indicates that the available adsorption sites on the material surface are approaching saturation; further increasing the solution concentration cannot markedly enhance the adsorption capacity but instead leads to a decline in removal efficiency because a unit mass of adsorbent is required to treat more pollutants [32].

The Freundlich and Langmuir isotherm models were employed to calculate the maximum removal capacity and to elucidate the underlying mechanism. The fitting results of the isothermal model are shown in Table 3. The fitting determination coefficient R^2^ values of the Langmuir and Freundlich models of LRMT are 0.983 and 0.915, respectively; the corresponding R^2^ values of MRMT are 0.971 and 0.910, and the R^2^ values of HRMT are 0.987 and 0.935. The fitting correlation coefficients of the Langmuir model for the three kinds of adsorbents are markedly higher than those of the Freundlich model, which indicates that the adsorption behavior of the adsorbents for the target pollutant is more in line with the uniform adsorption characteristics of monolayer adsorption than the multilayer heterogeneous adsorption process. The overall adsorption mechanism can be attributed to the exchange between solute molecules and adsorbate molecules in the pores of the adsorbent [33].

According to the Langmuir model parameters, the theoretical maximum adsorption capacity exhibited a trend of first increasing and then decreasing with the increase in the RM doping ratio. The Q_max_ value of MRMT reached 285.2 mg/g, which was higher than that of LRMT (275.1 mg/g) and HRMT (257.7 mg/g). This may be attributed to the fact that an excessively high RM content weakens the interfacial binding strength between the adsorbent and MB molecules. This complex structure–activity relationship highlights the importance of precisely regulating active components and support structures in the design of high-performance adsorbent materials. Similar phenomena have also been observed in other metal-doped porous carbon systems. Table 4 lists several other red mud-based adsorbents for direct comparison. For example, N-doping copolymer-derived hierarchical micro/mesoporous carbon achieved fine-tuning of pore structures by adjusting the precursor ratio, thereby optimizing its adsorption performance toward organic pollutants [34]. In addition, the separation factor (R_L_) values for the three materials ranged from 0.0167 to 0.0343, all falling within the favorable adsorption range of 0 to 1. The characteristic parameter 1/n values of the Freundlich model ranged from 0.168 to 0.229, all less than 1. Together, these results indicate that the adsorbents possess high affinity for MB, and the adsorption process is spontaneous, favorable, and readily occurring, corresponding to favorable adsorption.

### 3.4. Adsorption Thermodynamics

**Figure 5:**
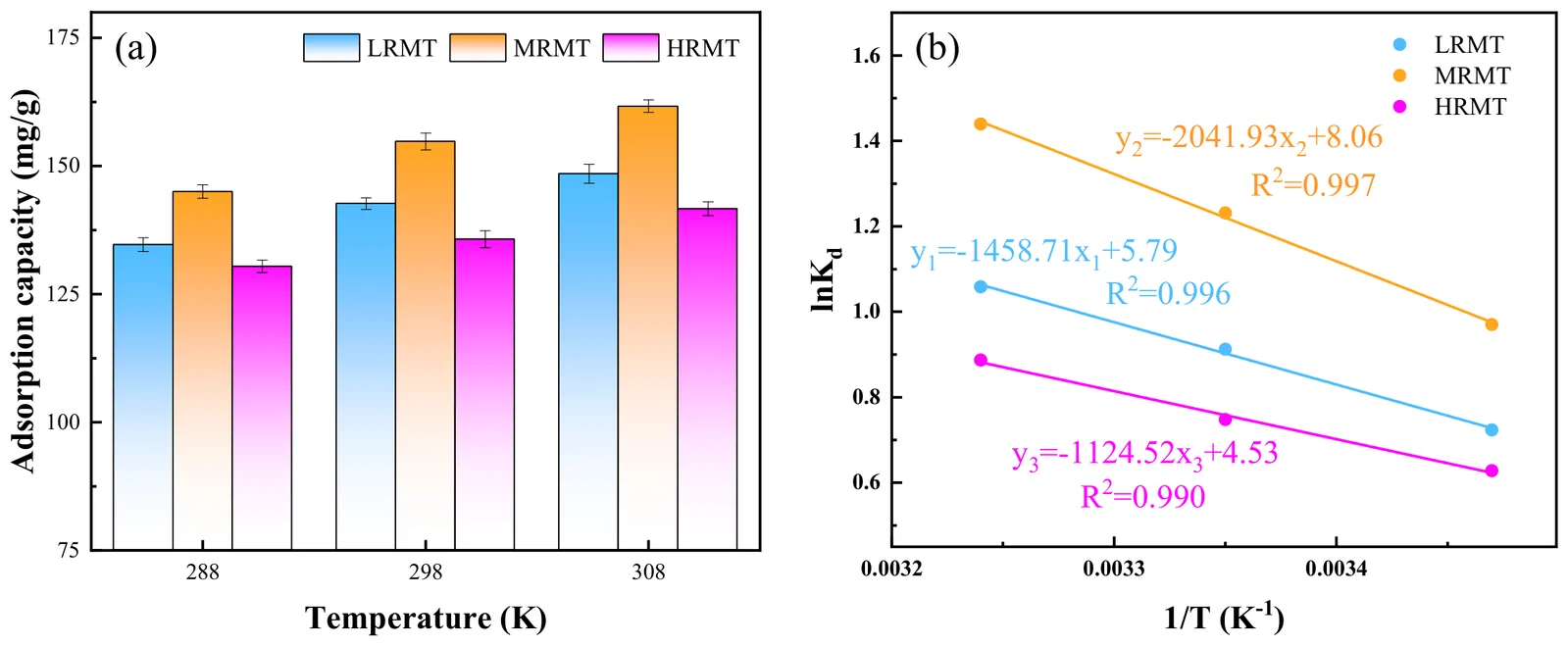
(**a**) Effect of temperature; (**b**) results obtained from the thermodynamic models.

As shown in Figure 5, the adsorption of MB on three kinds of composite biochars was studied at different temperatures to clarify its thermodynamic characteristics. In Figure 5a, it can be seen that for all three materials (LRMT, MRMT, and HRMT), the adsorption capacity increased with increasing temperature at the three tested temperatures. Furthermore, as shown in Table 5, the values of ΔH^0^ were 17.943 kJ/mol, 19.691 kJ/mol, and 12.123 kJ/mol, respectively, all of which were greater than zero, fully indicating that the adsorption processes are endothermic. Since 5 kJ/mol < ΔH^0^ < 40 kJ/mol, the processes can be classified as physical adsorption [37].

Within the experimental temperature range, the Gibbs free energy change (ΔG^0^) of the three kinds of composite biochars is negative, and its absolute value gradually increases with the increase in temperature. The results show that the pollutant adsorption process of biochar has spontaneous characteristics, and it belongs to the spontaneous adsorption reaction of endothermic and entropy increase [38].

The values of ΔS^0^ were positive and significantly large. This notable increase in entropy mainly originates from the increase in the overall disorder of the thermodynamic system [39]. In aqueous solution, MB^+^ ions are originally surrounded by one or more layers of highly ordered water molecules. Upon adsorption, MB^+^ ions are released from their hydration shells and bind to the adsorbent surface, resulting in the release of a large number of originally structured water molecules back into the bulk solution.

### 3.5. Influence of pH Value and Coexisting Ions

**Figure 6:**
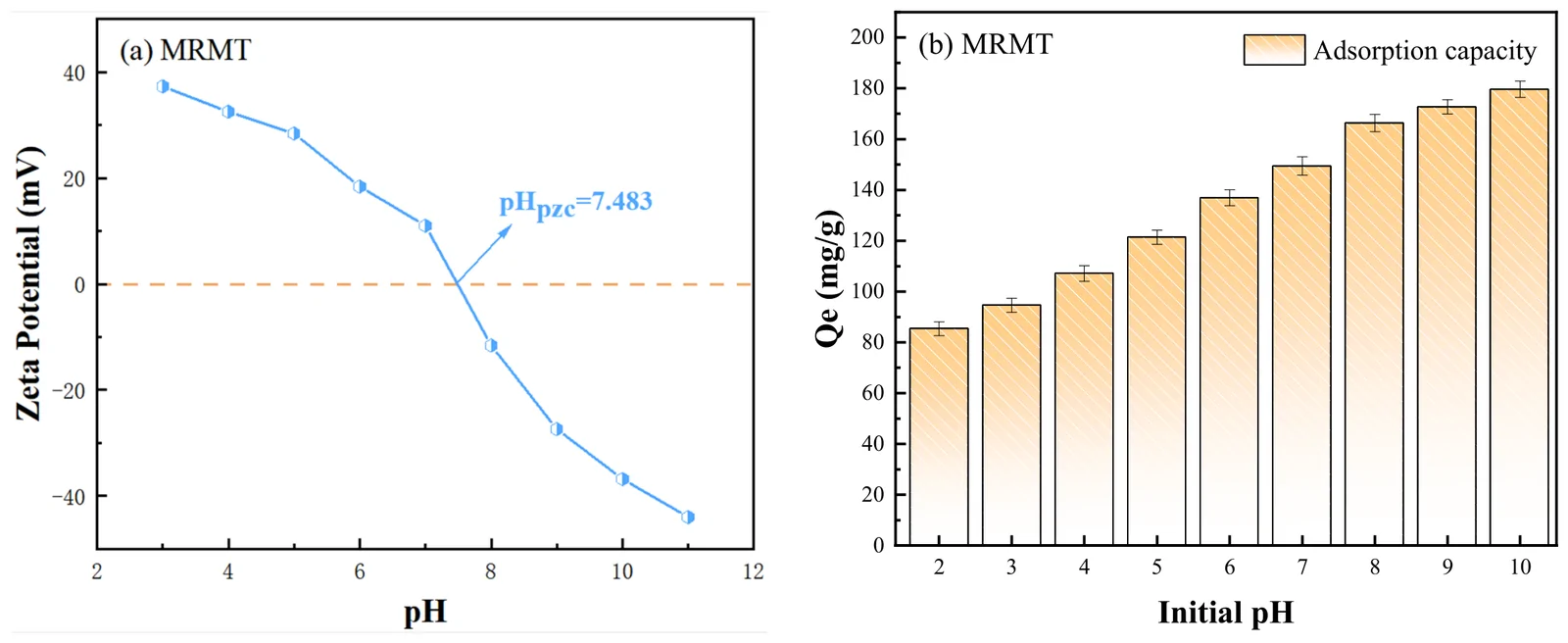

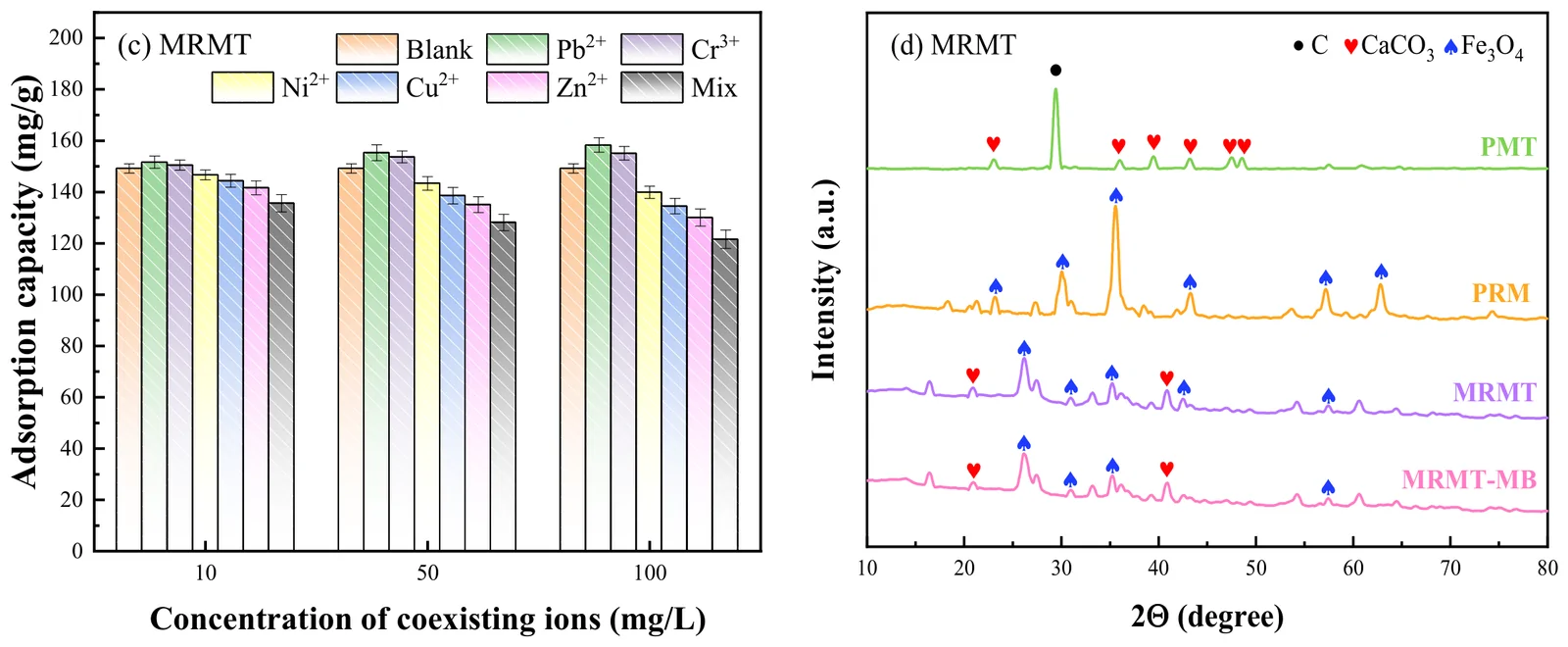
(**a**) Zeta potential analysis results; (**b**) effect of initial pH; (**c**) effect of coexisting ions; (**d**) XRD patterns of the samples.

Figure 6a shows the zeta potential variation of MRMT as a function of pH. The surface potential of the material gradually shifted from positive to negative with increasing solution pH, and the isoelectric point (pH_pzc_) was determined to be 7.483. When the solution pH was below pH_pzc_, the hydroxyl groups (–OH), iron–aluminum hydroxides (Fe–OH, Al–OH), and carbon-based oxygen-containing functional groups on the material surface underwent protonation. This process endowed MRMT with an overall positive charge, which created electrostatic repulsion with the positively charged cationic dye MB [40]. Moreover, at lower pH values, the high concentration of small-sized H^+^ ions preferentially occupied the active sites on the adsorbent surface [41]. Under these combined effects, the adsorption capacity decreased. When the pH exceeded pH_pzc_, the surface groups became deprotonated, rendering the surface negatively charged. An electrostatic attraction occurs between MB and the surface of the adsorbent, leading to an improvement in removal efficiency [42]. It is worth noting that when pH < pH_pzc_, MRMT still exhibits considerable adsorption capacity even in the presence of electrostatic repulsion. This indicates that non-electrostatic mechanisms (π–π, n–π, and pore filling) also play a significant role in the adsorption process.

Figure 6b presents the variation in adsorption capacity of MRMT for MB over the initial pH range of 2 to 10. The adsorption capacity was the lowest at pH = 2 (72.35 mg/g) and gradually increased as pH rose. The maximum capacity of 179.65 mg/g was obtained at pH = 10, which indicates that alkaline conditions favor MB adsorption [43]. This can be attributed to the electrostatic attraction between the negatively charged functional groups on the biochar and the positive charges of the cationic dye MB, particularly the positive charges on the amine groups and the nitrogen atoms within the rings [44].

Actual wastewater contains a large number of coexisting ions, which can exert varying degrees of influence on the adsorption of MB. Therefore, the effects of single-ion systems (Pb^2+^, Cr^3+^, Ni^2+^, Cu^2+^, Zn^2+^) and mixed-ion systems on the adsorption performance of MRMT were investigated at three concentration levels (10, 50, and 100 mg/L). The results in Figure 6c show that coexisting ions at low concentrations do not markedly inhibit MB adsorption. The adsorption capacity of MRMT can still reach more than 85% of the initial value. When the ion concentration reached 100 mg/L, an increase in the MB removal capacity was observed in the Pb^2+^ and Cr^3+^ systems. However, at high concentrations of Pb^2+^ and Cr^3+^, hydrolysis is likely to occur, and the possibility of MB being entrained in metal hydroxide precipitates cannot be ruled out. Therefore, there are two possible explanations for the observed enhancement: on the one hand, it may result from the formation of coordination complexes between metal cations and the adsorbent surface, creating additional adsorption sites; on the other hand, it is also possible that the removal of MB is due to entrainment by the hydrolysis and precipitation of metal oxides. In contrast, Ni^2+^ and Cu^2+^ can inhibit the formation of hydrogen bonds between MB molecules and functional groups on the surface of materials [45], thus suppressing MB adsorption. Although Cr^3+^ and Zn^2+^ have similar ionic radii, their mechanisms of action on the adsorption process differ significantly. Zn^2+^ has a moderate charge density [46]. It cannot form effective coordination structures or activate intrinsic active sites [47], thus hindering the adsorption process of MRMT. Nevertheless, MB adsorption on MRMT also relies on mechanisms that are immune to cation competition. Typical examples include π–π stacking interactions with the carbon skeleton and physical pore adsorption. Therefore, the inhibitory effects of coexisting ions are limited. The material still maintains a relatively high adsorption capacity even at high concentrations of coexisting ions. In the mixed coexisting ion system, multiple cations compete for the negatively charged surface sites simultaneously. Although the adsorption capacity declined more obviously than in the single-ion systems, it remained at a relatively high level overall. This finding demonstrates that MRMT had stable adsorption performance even in complex water matrices with multiple coexisting ions.

### 3.6. Adsorption Mechanism

#### 3.6.1. XRD

As shown in Figure 6d, the XRD patterns of PMT, PRM, MRMT, and MRMT-MB clearly reflect the regulation of the raw material ratio and methylene blue adsorption on the crystalline phase structure of the materials. PMT exhibits the strongest characteristic peak at 2θ ≈ 29.3°, originating from the amorphous structure of the biochar [30], while the diffraction peaks at 23.0°, 35.9°, 39.4°, 43.2°, and 47.5° correspond to CaCO_3_ derived from the native calcium in the biomass feedstock. PRM shows various diffraction peaks corresponding to Fe-containing phases at 29.9°, 35.6°, and 43.2°, which may be attributed to Fe_3_O_4_ in the pyrolyzed red mud [48]. MRMT exhibits partial diffraction peaks of both Fe_3_O_4_ and CaCO_3_, and the carbon phase peaks are largely retained, indicating that the composite system features a coexistence of an amorphous carbon phase and polycrystalline mineral phases. Comparing the pattern of MRMT-MB after adsorption with that of MRMT before adsorption, it can be seen that the 2θ positions and peak patterns of all characteristic diffraction peaks are very similar before and after adsorption, with no new sharp diffraction peaks appearing and no original characteristic peaks disappearing. However, after adsorption, the intensities of all diffraction peaks decreased to varying degrees, with the magnetite characteristic peaks at medium–high angles showing more significant attenuation because of the dilution of the crystalline phases, indicating that MB was successfully loaded onto the adsorbent.

#### 3.6.2. FTIR

**Figure 7:**
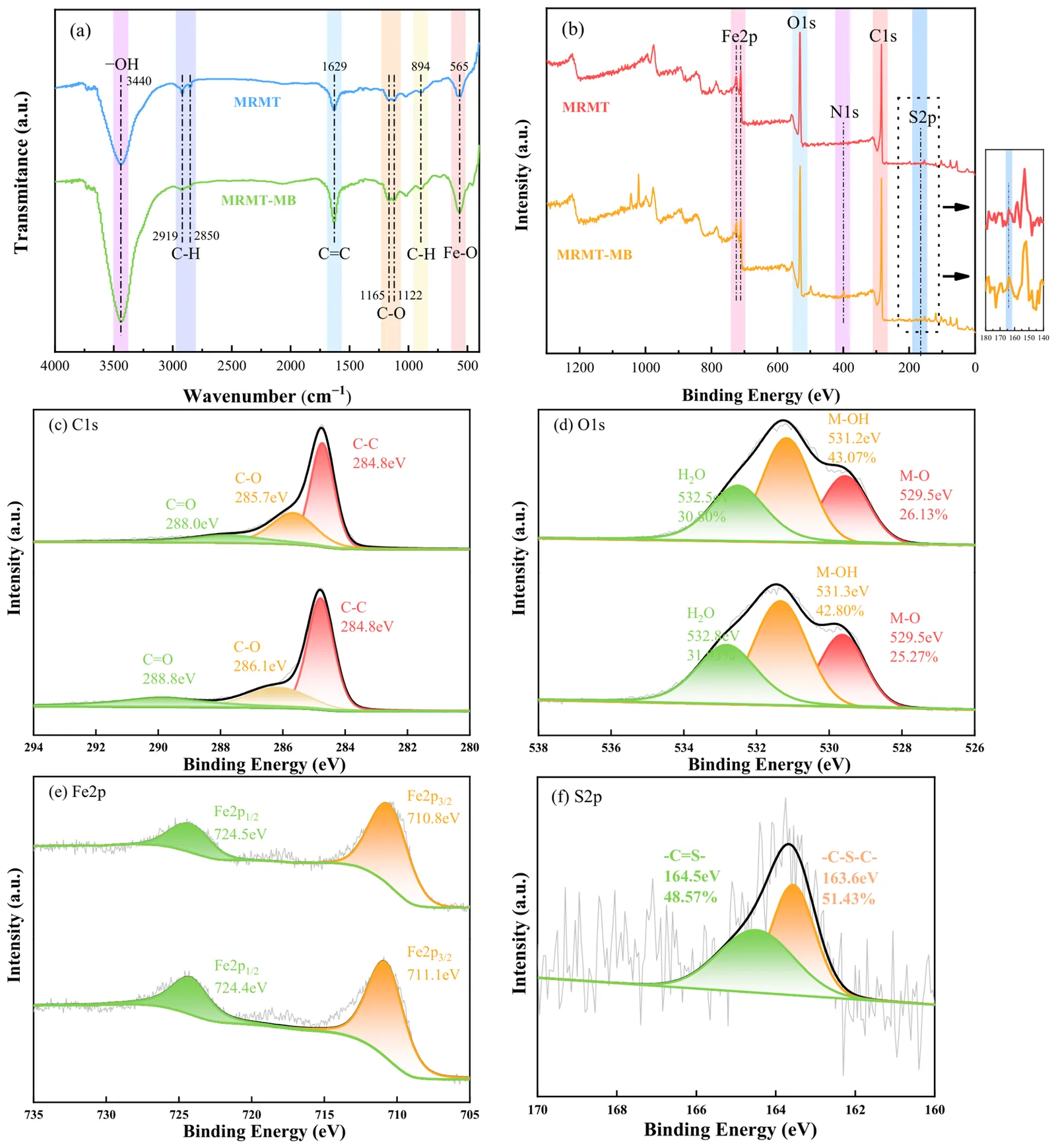
FTIR spectrum of MRMT and XPS spectra of MRMT before and after adsorption.

Figure 7a shows the FTIR spectra of MRMT before and after MB adsorption. The broad and strong absorption band at 3440 cm^−1^ is attributed to the stretching vibration of surface hydroxyl groups (–OH) [49], originating from the bound water and hydroxyl groups on the carbon-based oxygen-containing functional groups and the surface of iron–aluminum oxides. The absorption peak at 1629 cm^−1^ corresponds to the vibration of the C=C skeleton in the graphitized aromatic structure [30]. The weak peaks at 2919 cm^−1^ and 2850 cm^−1^ are assigned to the asymmetric and symmetric stretching vibrations of aliphatic C–H bonds. The peaks at 1165 cm^−1^ and 1122 cm^−1^ are attributed to the stretching vibrations of C–O bonds, and these characteristic peaks correspond to typical functional groups in the biochar structure [50]. The peak at 894 cm^−1^ is due to the out-of-plane bending vibration of aromatic C–H bonds. The characteristic peak at 565 cm^−1^ originates from the stretching vibration of Fe–O in Fe_3_O_4_ [51], which is consistent with the XRD phase analysis results described above. Comparing the spectra of MRMT-MB after adsorption with those before adsorption, it can be seen that the positions and intensities of all characteristic peaks changed to varying degrees, with the intensities at 1629 cm^−1^ and 3440 cm^−1^ being notably enhanced after adsorption. The enhancement at 1629 cm^−1^ is due to the vibrations of C=N and C=C in the MB heterocyclic ring, while that at 3440 cm^−1^ is attributed to the hydroxyl groups (OH) and the nitrogen atoms of unsaturated dimethylamine groups involved in binding to the heterocyclic nitrogen atoms of MB [52].

#### 3.6.3. XPS

To further understand the adsorption mechanism of MRMT toward MB, XPS analysis was conducted on the samples before and after adsorption. As shown in Figure 7b, after MB adsorption, distinct N1s and S2p characteristic peaks appeared in the spectrum of MRMT. Since N and S are the characteristic constituent elements of methylene blue molecules (molecular formula: C_16_H_18_ClN_3_S), this confirms the successful loading of MB. Figure 7c presents the peak-fitting results of C1s. The C1s spectrum of MRMT can be deconvoluted into three characteristic peaks corresponding to C–C, C–O–C, and C=O. These groups represent the main surface functional groups in biochar. The result indicates that the adsorbent possesses an aromatic structure capable of participating in interactions through electron pair donation and π-electrons, thereby promoting MB removal [49]. After MB adsorption, the binding energies of C–O and C=O shifted from 285.7 eV and 288.0 eV to 286.1 eV and 288.8 eV, respectively. These shifts are attributed to the interactions between the chemical groups on the adsorbent and MB molecules [53]. In the O1s peak-fitting results (Figure 7d), the binding energies at 532.5 eV, 531.2 eV, and 529.5 eV correspond to –CO–NH–, C–O–C, and C–O–H, respectively. After adsorption, the binding energy of –CO–NH– shifted slightly to 532.8 eV, and its relative proportion increased from 30.80% to 31.93%, while that of C–O–H decreased from 26.13% to 25.27%. These changes in absorption peaks are ascribed to the interactions between electron donors involved in the adsorption process and the positively charged amino groups of MB [53]. In addition, the inorganic oxygen atoms on the adsorbent surface can serve as electron donors that participate in n–π interactions with the aromatic rings of MB [54]. In Figure 7e, the two main peaks in the Fe2p spectrum correspond to Fe 2p_1_/_2_ and Fe 2p_3_/_2_, indicating the coexistence of Fe^3+^ and Fe^2+^ on the adsorbent [55]. This finding further confirms the presence of magnetite. After adsorption, the binding energies shifted from 724.5 eV and 710.8 eV to 724.4 eV and 711.1 eV, respectively, indicating that Fe participated in the adsorption reaction of MB. After the adsorption is complete, the characteristic peaks appear in the S2p spectrum of the sample (Figure 7f) at 164.5 eV and 163.6 eV. These peaks belong to -C=S- and -C-S-C- in the MB molecule, respectively [56].

Combining the FTIR and XPS characterization results, the adsorption of MB onto MRMT is a process that involves multiple adsorption mechanisms. These mechanisms mainly include pore filling, π–π stacking, n–π interactions, electrostatic attraction, and hydrogen bonding [27]. Multiple adsorption mechanisms are the core reason for the excellent adsorption performance of MRMT toward MB.

### 3.7. Recovery Capacity

**Figure 8:**
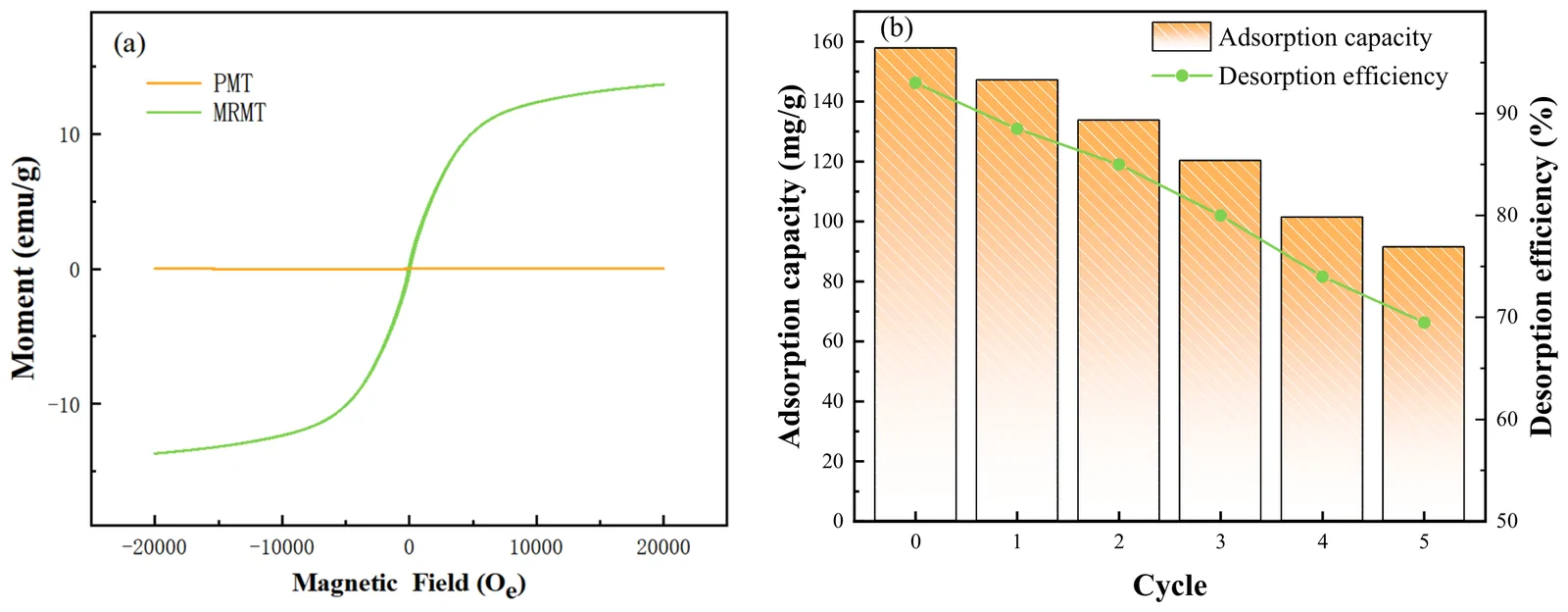
(**a**) VSM curve; (**b**) recycling experimental results.

The magnetic behavior of the composite biochars was evaluated by VSM characterization, as shown in Figure 8a. The results showed that the magnetization intensity of PMT remained at zero throughout, indicating that it is a typical non-magnetic material and is difficult to recover efficiently from wastewater systems by physical separation. In contrast, MRMT exhibited a clear hysteresis loop. According to previous studies, such magnetic materials can demonstrate excellent stability and good recyclability during the adsorption of MB [57], further indicating that MRMT possesses potential as an efficient adsorbent for MB removal. As can be seen in the figure, the hysteresis loop is S-shaped and passes through the origin, with no significant remanent magnetization or coercivity, indicating that the composite biochar exhibits superparamagnetism [58]. The saturation magnetization of the adsorbent is 13.66 emu/g, and high-efficiency magnetic separation can be achieved under the condition of an external magnetic field. This shows that MRMT has excellent magnetic response performance and can simply and quickly complete solid–liquid separation and adsorbent recovery [59].

Through consecutive adsorption–desorption cycling experiments, the reusability and structural stability of the material were systematically evaluated. The adsorption capacity of MRMT toward MB after five cycles is shown in Figure 8b. After the first cycle, the adsorption capacity of MB showed no significant decrease, remaining at 93% of its original value. After five cycles, the adsorption capacity gradually decreased to 57% of the original adsorption capacity. The gradual decline in adsorption efficiency may be attributed to some dye molecules remaining adsorbed on the surface of the adsorbent [60]. After five consecutive adsorption–desorption cycles, the desorption efficiency of MRMT for methylene blue can still be maintained above 68%, which proves that the adsorbent has excellent structural stability and recyclable performance.

## 4. Conclusions

In this study, a magnetic composite adsorbent (MRMT) was prepared by one-step pyrolysis with industrial solid waste red mud and agricultural waste Medulla tetrapanacis as raw materials, achieving the dual goals of solid waste recycling and methylene blue wastewater purification. The material exhibits a microporous–mesoporous hierarchical pore structure. The active sites of Fe/Al minerals introduced by red mud, along with oxygen-containing functional groups, are the key contributors to its adsorption performance, while its specific surface area plays only a supporting role in mass transfer. Under neutral conditions, MRMT demonstrates excellent methylene blue adsorption capacity (285.2 mg/g) while also exhibiting good magnetic separation recovery capacity and some potential for recycling. Furthermore, because the raw materials for this adsorbent are inexpensive and environmentally friendly, it has the potential to reduce waste generation and be reused. Overall, the present study not only provides a new approach for the high-value utilization of industrial red mud and agricultural Medulla tetrapanacis but also offers a magnetic recovery adsorption material with application potential, which is highly consistent with the concepts of green sustainable development and circular economy.

## 5. Limitations and Future Directions

The MRMT composite biochar prepared in this study exhibited good methylene blue adsorption, magnetic separation, and resistance to ionic interference; however, it still has certain limitations.

(1) The adsorption tests did not use actual dyeing and printing wastewater; adsorption performance may vary significantly in real water bodies.
(2) In adsorption models, linearization alters the error structure, introducing systematic biases in the fitting parameters; furthermore, the use of a relatively low adsorbent concentration of 0.5 g/L in isothermal experiments tends to slightly increase the apparent maximum Langmuir adsorption capacity.
(3) Since PMT is non-magnetic, no adsorption tests were conducted on it; although it has the highest specific surface area, there was no control group for comparison.
(4) The coexisting ion experiments lacked a blank control group; the hydrolysis and precipitation of metal ions may introduce uncertainty in the interpretation of the results.
(5) The absence of scale-up experiments and techno-economic analysis means that the actual economic benefits of the complete process remain to be verified.

Therefore, in future research, real textile printing–dyeing wastewater should be employed to evaluate treatment performance under complex water matrix conditions and explore the competitive inhibitory effects induced by salts and textile-relevant surfactants. We also suggest investigating MB adsorption performance under high adsorbent dosage conditions and carrying out fitting via nonlinear regression. Moreover, supplementary control adsorption experiments of PMT should be performed, and blank control groups for coexisting ion systems need to be established to distinguish real adsorption from co-precipitation–entrainment artifacts. Meanwhile, pyrolysis and regeneration processes should be optimized, and pilot-scale trials and full-process techno-economic assessment should be implemented to promote the practical application of this type of solid-waste-based adsorbent.

## Figures and Tables

**Table 1:** Total pore volume, average pore diameter, and BET specific surface area of the samples.

Sample	BET Surface Area (m^2^/g)	Total Pore Volume (cm^3^/g) *	Average Pore Size (nm)
PMT	374.8	0.2760	2.946
PRM	4.707	0.0327	27.82
LRMT	262.2	0.4378	5.045
MRMT	248.5	0.3981	6.407
HRMT	198.0	0.3307	8.843

* The average pore size is equal to 4 V/A according to the BET.

**Table 2:** The adsorption kinetic data obtained.

Adsorbent	T (K)	PFO Model			PSO Model		
		K_1_ (1/min)	Q_e_ (mg/g)	R^2^	K_2_ (g/mg × min)	Q_e_ (mg/g)	R^2^
LRMT	298	0.205	134.2	0.943	1.010 × 10^−3^	145.7	0.997
MRMT	298	0.297	149.2	0.937	1.370 × 10^−3^	157.9	0.992
HRMT	298	0.198	130.3	0.937	9.740 × 10^−4^	139.0	0.994

**Table 3:** Adsorption isotherm data for LRMT, MRMT, and HRMT.

Adsorbent	T (K)	Langmuir Model				Freundlich Model		
		Q_max_ (mg/g)	K_L_ (L/mg)	R_L_	R^2^	K_F_ ((mg/g)(L/mg)^1/n^)	1/n	R^2^
LRMT	298	275.1	0.142	0.0310	0.983	93.52	0.218	0.915
MRMT	298	285.2	0.268	0.0167	0.971	129.3	0.168	0.910
HRMT	298	257.7	0.128	0.0343	0.987	80.97	0.229	0.935

**Table 4:** Comparison of the MB adsorption performance of different red mud-based adsorbents.

Adsorbent	Raw Materials	Preparation Conditions	Dosage (g/L)	C_0_range (mg/L)	pH	T (K)	Equilibrium Time (min)	Q_max_ (mg/g)	Reference
MRMT	Red mud, Medulla tetrapanacis	Pyrolysis, 800 °C, 2 h	0.5	60–220	7	298	480	285.2	this work
RFBC	Red mud, food waste compost	PTFE reactor at 180 °C, 20 h	2	100–500	11	298	720 min	124.06	[18]
MHC	Red mud, peanut shell	Teflon reactor 200 °C, 12 h	1	20–160	10	298	120 min	123.26	[19]
ARM-200	Red mud	Pyrolysis, 200 °C, 12 h	1	100–300	7	298	120 min	274	[35]
MRM	Red mud	Teflon reactor at 140 °C, 12 h	3	100–1000	7	298	300 min	280.18	[36]

**Table 5:** Thermodynamic model data for LRMT, MRMT, and HRMT.

Adsorbent	ln K_d_			ΔG_0_ (kJ/mol)			The Standard Error		ΔH_0_ (kJ/mol)	ΔS_0_ (J/(mol·K))
	288 K	298 K	308 K	288 K	298 K	308 K	Slope	Intercept		
LRMT	16.92	17.20	17.42	−40.54	−42.61	−44.61	66.32	0.222	17.943	203.028
MRMT	17.58	17.83	18.13	−42.10	−44.18	−46.43	167.32	0.561	19.691	214.335
HRMT	16.86	17.10	17.28	−40.37	−42.37	−44.25	64.79	0.217	15.123	192.719

## Data Availability

The original contributions presented in this study are included in this article. Further inquiries can be directed to the corresponding authors.

## References

[1] Siddique H.M.A., Kiani A.K. (2020). Industrial pollution and human health: Evidence from middle-income countries. Environ. Sci. Pollut. Res..

[2] Chen J., Zhang J., Chen X., Zhu Y., Liu X., Ma S., Huang A., Wu Y., Lin F., Zhou Y. (2026). Metal-organic frameworks for catalytic degradation of emerging dye contaminants in wastewater: A critical review of material design, reaction mechanisms, and engineering application. Results Eng..

[3] Bourachdi S.E., Amri A.E., Ayub A.R., Moussaoui F., Rakcho Y., Ouadrhiri F.E., Adachi A., Bouzid T., Herrera-Melián J.A., Lahkimi A. (2025). Synthesis and characterization of avocado pit activated carbon-incorporated chitosan composite beads for harnessing methylene blue adsorption: DFT insights and box-behnken design optimization. J. Taiwan Inst. Chem. Eng..

[4] Yang Y., Zeng X., Zeng Z., Wang L., Zhu P., Tang L., Zhu L. (2026). Efficient removal of dyes and antibiotics from oily wastewater by polypropylene/ZIF-67 composite membranes with underwater superoleophobicity and persulfate advanced oxidation processes. Sep. Purif. Technol..

[5] Elsayed E., Grosso V., Fuoco A., Kourmentza K., Tokay B. (2026). ZIF-67/Bacterial cellulose (BC) membranes for the removal of dyes and oil/water separation. Sep. Purif. Technol..

[6] Ma J., Wang H., Yin Z., Kong Y., Chang Y., Nie Y. (2026). Effective removal of methyl blue (MB) via different *Conidiobolus coronatus* strains isolated from China: Adsorption and degradation studies. Sep. Purif. Technol..

[7] Obayomi K.S., Mustapha L.S., Falowo O.A., Obayomi O.V., Dembaremba T.O., Enidiok E.S., Zhang J. (2026). Adsorption activity of cerium-magnesium ferrite nanoparticles on woodchip-derived biochar for enhanced organic pollutants removal. Desalination.

[8] Anastopoulos I., Ahmed M.J., Ojukwu V.E., Danish M., Stylianou M., Ighalo J.O. (2024). A comprehensive review on adsorption of Reactive Red 120 dye using various adsorbents. J. Mol. Liq..

[9] Tan X., Liu Y., Zeng G., Wang X., Hu X., Gu Y., Yang Z. (2015). Application of biochar for the removal of pollutants from aqueous solutions. Chemosphere.

[10] Li X., Wang C., Zhang J., Liu J., Liu B., Chen G. (2020). Preparation and application of magnetic biochar in water treatment: A critical review. Sci. Total Environ..

[11] Krammer K.B., Rios L.C., Santos B.R., Munhoz I.S., Müller D.G., Ribeiro E.S., Maciel J.V., Dias D., Oreste E.Q. (2026). Recent progress in the development and application of magnetic biochar for the adsorption of potentially toxic metals from aquatic systems: A review. Environ. Surf. Interfaces.

[12] Iwuozor K.O., Okoro H.K., Adeniyi A.G. (2026). A Critical Review on Metal/Metal Oxide-Modified Sugarcane Biochar. Results Eng..

[13] Li Q., Wei G., Zhang L., Li Z., Li J. (2023). Activation of peroxymonosulfate by a waste red mud-supported Co_3_O_4_ quantum dots under visible light for the degradation of levofloxacin. Chem. Eng. J..

[14] Hou K., Chen P., Chen W., Li Y., Yang Y., Zhang C. (2025). Activated red mud-based low-carbon cementitious composite: Hydration mechanism, mechanical properties, and carbon emissions. Sustain. Chem. Pharm..

[15] Zhao X., Liao Z., Zhao Q., Yang M., Li D., Zhang K., Wang X., Zhang H., Zheng B. (2025). A low cost magnetic biochar manufactured solely from solid wastes by one-step pyrolysis for removal of tetracycline. Sci. Rep..

[16] Zhang L., Li W., Cao H., Hu D., Chen X., Guan Y., Tang J., Gao H. (2019). Ultra-efficient sorption of Cu^2+^ and Pb^2+^ ions by light biochar derived from *Medulla tetrapanacis*. Bioresour. Technol..

[17] Liu Z., Dai C., Hu Y., Zhang J., Wang Y., Zhang L., Ye P., Zhang T., Guan Y., Gao H. (2025). Efficient removal of Cu^2+^ on *Medulla Tetrapanacis*-based modified biochar: Adsorption performance, mechanisms and recycling for fabrication of supercapacitor electrodes. Chem. Eng. J..

[18] Li P., Yang Z., Li Y., Tian Y. (2024). Preparation of efficient adsorbent for methylene blue and Congo red by co-hydrothermal of red mud with food waste compost. Desalin. Water Treat..

[19] Qiu J., Yang J., Cui K., Cao X., Lyu J., Liu H., Wang J., Wang G., Liu X. (2025). Synergistic waste valorization: One-step hydrothermal synthesis of magnetic hydrochar from red mud and peanut shells for effective methylene blue adsorption. Mater. Chem. Phys..

[20] Thines K.R., Abdullah E.C., Mubarak N.M., Ruthiraan M. (2017). Synthesis of magnetic biochar from agricultural waste biomass to enhancing route for waste water and polymer application: A review. Renew. Sustain. Energy Rev..

[21] Tran H.N. (2022). Improper estimation of thermodynamic parameters in adsorption studies with distribution coefficient KD (qe/Ce) or freundlich constant (KF): Considerations from the derivation of dimensionless thermodynamic equilibrium constant and suggestions. Adsorpt. Sci. Technol..

[22] Qin J., Wang X., Deng M., Li H., Lin C. (2022). Red mud-biochar composites (co-pyrolyzed red mud-plant materials): Characteristics and improved efficacy on the treatment of acidic mine water and trace element-contaminated soils. Sci. Total Environ..

[23] Qiu Y., Yuan H., Yang X., Leng Y., Liu X. (2025). Enhanced degradation of levofloxacin hydrochloride synergistically with hydroxylamine hydrochloride via Fe_3_O_4_-loaded *Medulla Tetrapanacis* residue in a Fenton-like process. J. Water Process Eng..

[24] Matthias Thommes K.K.A.V. (2015). Physisorption of gases, with special reference to the evaluation of surface area and pore size distribution (IUPAC Technical Report). Pure Appl. Chem..

[25] Lin J.E., Guo H., Zhao Z., Kang H., Guo Y., Peng T., Li K., Yan L. (2025). Lanthanum-doped mesoporous magnetic carbon microspheres derived from Camellia oleifera shell biomass for phosphate removal from industrial wastewater. Sep. Purif. Technol..

[26] Liu Z., Hu Y., Zhang J., Guan Y., Zhang L., Ye P., Zhang T., Huang X., Wang M., Gao H. (2023). Enhanced adsorption of Congo red from urea/calcium chloride co-modified biochar: Performance, mechanisms and toxicity assessment. Bioresour. Technol..

[27] Liu J., Lin Q., Gao J., Jia X., Cai M., Liang Q. (2023). Adsorption properties and mechanisms of methylene blue and tetracycline by nano-silica biochar composites activated by KOH. Chemosphere.

[28] Li X., Xu J., Luo X., Shi J. (2022). Efficient adsorption of dyes from aqueous solution using a novel functionalized magnetic biochar: Synthesis, kinetics, isotherms, adsorption mechanism, and reusability. Bioresour. Technol..

[29] Xiong D., Dong X., Zhang H., Han T., Zhang L., Qiao Y., Cen Q. (2026). Synergistic valorisation of red mud and biomass: Enhanced dye adsorption and environmental safety via Co-Pyrolysis for sustainable waste management. J. Environ. Chem. Eng..

[30] Azim E.A., Samy M., Hanafy M., Mahanna H. (2024). Novel mint-stalks derived biochar for the adsorption of methylene blue dye: Effect of operating parameters, adsorption mechanism, kinetics, isotherms, and thermodynamics. J. Environ. Manag..

[31] Xu Q., Chen Z., Wu Z., Xu F., Yang D., He Q., Li G., Chen Y. (2019). Novel lanthanum doped biochars derived from lignocellulosic wastes for efficient phosphate removal and regeneration. Bioresour. Technol..

[32] Novais R.M., Ascensão G., Tobaldi D.M., Seabra M.P., Labrincha J.A. (2018). Biomass fly ash geopolymer monoliths for effective methylene blue removal from wastewaters. J. Clean. Prod..

[33] Liu Z., Zhang J., Zhang L., Guan Y., Ji H., Zhang Y., Gao H. (2023). Efficient removal of Congo red and methylene blue using biochar from Medulla Tetrapanacis modified by potassium carbonate. Bioresour. Technol..

[34] Liu Y., Qi R., Ge Z., Zhang Y., Jing L., Li M. (2021). N-doping copolymer derived hierarchical micro/mesoporous carbon: Pore regulation of melamine and fabulous adsorption performances. J. Taiwan Inst. Chem. Eng..

[35] Hu Z.-P., Gao Z.-M., Liu X., Yuan Z.-Y. (2017). High-surface-area activated red mud for efficient removal of methylene blue from wastewater. Adsorpt. Sci. Technol..

[36] Hu H., Yang Y., Zhou G., Wang N., Gu H. (2025). Hydrothermal chemical modification of red mudfor efficient adsorption of methylene blue. Environ. Technol..

[37] Soares S.F., Pereira B., Santos J., Trindade T., Barreto Â., Maria V.L., Daniel-Da-Silva A.L. (2025). Charge-selective magnetic bionanosorbents for multi-dye wastewater treatment: Performance, regeneration and ecotoxicity assessment. Sep. Purif. Technol..

[38] Yang J., Ma X., Xiong Q., Zhou X., Wu H., Yan S., Zhang Z. (2023). Functional biochar fabricated from red mud and walnut shell for phosphorus wastewater treatment: Role of minerals. Environ. Res..

[39] Mensah K., Samy M., Mahmoud H., Fujii M., Shokry H. (2022). Rapid adsorption of sulfamethazine on mesoporous graphene produced from plastic waste: Optimization, mechanism, isotherms, kinetics, and thermodynamics. Int. J. Environ. Sci. Technol..

[40] Zhang H., Peng B., Liu Q., Wu C., Li Z. (2022). Preparation of porous biochar from heavy bio-oil for adsorption of methylene blue in wastewater. Fuel Process. Technol..

[41] Wang J., Yu J., Li M., Zhang Y. (2024). Synthesis of sodium polystyrene sulfonate resins for the removal of methylene blue from wastewater. J. Taiwan Inst. Chem. Eng..

[42] Abollino O., Aceto M., Malandrino M., Sarzanini C., Mentasti E. (2003). Adsorption of heavy metals on Na-montmorillonite. Effect of pH and organic substances. Water Res..

[43] Shao X., Zhong J., Xu D., Ren X., Zhou H., Wang C. (2026). Synergistic preparation of magnetic biochar from sewage sludge and iron tailings for dye adsorption: Contrasting effect of hydrothermal carbonization and pyrolysis. J. Water Process Eng..

[44] Hamou A.B., Elamraoui S., Escudero-Curiel S., Chaoui A., Farsad S., Amjlef A., Nouj N., Saghir Y., Benafqir M., Sanromán Á. (2025). Enhancing surface area of HNO3-activated digestate-derived biochar for methylene blue adsorption: RSM-CCD optimization, DFT-MD calculations, and mechanistic study. Appl. Surf. Sci..

[45] Qiu B., Shao Q., Shi J., Yang C., Chu H. (2022). Application of biochar for the adsorption of organic pollutants from wastewater: Modification strategies, mechanisms and challenges. Sep. Purif. Technol..

[46] Zhang L., Liu B., Jiao F. (2025). Adsorption mechanism of first-row transition metal ions (Fe, Co, Ni, Cu, Zn) on the muscovite (001) surface: A DFT study. Comput. Theor. Chem..

[47] Han L., Wang T., Gong J., Li X., Ji Y., Wang S. (2022). Multi-hydroxyl containing organo-vermiculites for enhanced adsorption of coexisting methyl blue and Pb(II) and their adsorption mechanisms. Colloids Surf. A Physicochem. Eng. Asp..

[48] Chen H., Zhao K., Wang Y., Liu Y., Zhang X. (2026). Red mud-derived functional materials: Engineering strategies, mechanistic insights, and synergistic applications in water, soil, and atmospheric restoration. J. Ind. Eng. Chem..

[49] Zheng Y., Wang J., Li D., Liu C., Lu Y., Lin X., Zheng Z. (2021). Insight into the KOH/KMnO_4_ activation mechanism of oxygen-enriched hierarchical porous biochar derived from biomass waste by in-situ pyrolysis for methylene blue enhanced adsorption. J. Anal. Appl. Pyrol..

[50] Zhou P., Li X., Zhou J., Peng Z., Shen L., Li W. (2023). Insights of the adsorption mechanism of methylene blue on biochar from phytoextraction residues of *Citrus aurantium* L.: Adsorption model and DFT calculations. J. Environ. Chem. Eng..

[51] Mortada W.I., Ghaith M.M., Khedr N.E., Ellethy M.I., Mohsen A.W., Shafik A.L. (2024). Mesoporous magnetic biochar derived from common reed (*Phragmites australis*) for rapid and efficient removal of methylene blue from aqueous media. Environ. Sci. Pollut. Res..

[52] Ovchinnikov O.V., Evtukhova A.V., Kondratenko T.S., Smirnov M.S., Khokhlov V.Y., Erina O.V. (2016). Manifestation of intermolecular interactions in FTIR spectra of methylene blue molecules. Vib. Spectrosc..

[53] Ma W., Liu X., Lu H., He Q., Ding K., Wang X., Wang W., Guo F. (2023). Chitosan-based composite hydrogel with a rigid-in-flexible network structure for pH-universal ultra-efficient removal of dye. Int. J. Biol. Macromol..

[54] Yang C., Wu H., Cai M., Li Y., Guo C., Han Y., Zhang Y., Song B. (2023). Valorization of food waste digestate to ash and biochar composites for high performance adsorption of methylene blue. J. Clean. Prod..

[55] Hien D.T., Cong P.T., Van Anh N.T., Dat T.D. (2026). Properties of CoFe_2_O_4_/biochar from orange peel: Exploration of methylene blue adsorption mechanism and biocompatibility. Nano-Struct. Nano-Objects.

[56] Sarkar A.K., Bediako J.K., Choi J., Yun Y. (2019). Functionalized magnetic biopolymeric graphene oxide with outstanding performance in water purification. NPG Asia Mater..

[57] Mian M.M., Liu G. (2019). Sewage sludge-derived TiO2/Fe/Fe3C-biochar composite as an efficient heterogeneous catalyst for degradation of methylene blue. Chemosphere.

[58] Zhong J., Luo B., Feng Y., Ying G., Chen Z. (2026). Enhanced removal of tetracycline via defect-rich iron-biochar composite in typical soil: Moisture regulation and oxidation pathway. Appl. Surf. Sci..

[59] Yin S., Wu Z., Li W., Guo B., Guo X., Meng Y., Chen L., Bai Z., Yu Q., Zhou Y. (2025). Facile synthesis of magnetic Ce-Fe_3_O_4_/banana peel biochar nanocomposite by in situ self-activation for enhanced Rhodamine B removal: Performance and mechanism. Colloids Surf. A Physicochem. Eng. Asp..

[60] Vishwakarma D., Rawat S., Kannaujiya D., Awasthi S., Shikha (2026). Synthesis of a novel sodium alginate/bentonite based biocomposite in combination with iron modified walnut shell (*Juglans regia*) biochar for the enhanced adsorption of methylene blue dye: Synthesis, characterization, adsorption, isotherms, and kinetics. Environ. Sustain..

